# Classification of Hallucal Sesamoid Bone Correlated with Hallux Valgus Severity

**DOI:** 10.1155/2020/9658916

**Published:** 2020-06-26

**Authors:** Lei Zhang, Junqiu Wang, Jiaju Liu, Jiangqin Luo

**Affiliations:** ^1^Department of Orthopedics, Affiliated Traditional Chinese Medicine Hospital of Southwest Medical University, Luzhou, China 646000; ^2^Academician Workstation in Luzhou, Luzhou, China 646000; ^3^Center for Orthopedic Diseases Research, Affiliated Traditional Chinese Medicine Hospital of Southwest Medical University, Luzhou, China 646000; ^4^Clinical Base of Affiliated Traditional Chinese Medicine Hospital of Southwest Medical University, Guangdong Province Medical 3D Printing Application Transformation Engineering Technology Research Center, Guangdong Province, China 510000; ^5^School of Chinese and Western Clinical Medicine, Southwest Medical University, Luzhou, China 646000; ^6^Market Supervision Administration of Luzhou City of Sichuan Province, Kangcheng Road, Jiangyang District, Luzhou City, Sichuan Province, China 646000; ^7^Department of Operation Theatre, Affiliated Traditional Chinese Medicine Hospital of Southwest Medical University, Luzhou, China 646000

## Abstract

The hallucal sesamoid bones (HSBs), having an important role in reducing load per unit area on the first metatarsal head, can be injured commonly which also affected the first metatarsophalangeal joint and the surrounding structure. Meanwhile, differences among each HSB type may be a major factor affecting the occurrence and development of HV. So far, many researchers had learned that there are three different conditions in hallucal sesamoid bone affecting the choice of clinical surgery corresponding to different solutions in clinic. Thus, it is necessary to study the anatomical morphological characteristics of the HSB which can be helpful in clinical diagnosis and treatment, especially hallux valgus (HV). 150 X-ray and three-dimensional (3D) computed tomographic (CT) images consist of 72 left and 78 right metatarsals were applied in this anatomic study between two variables and showed by a simple scatter plot. The first metatarsophalangeal joint is divided into four different types: type I (no HSB, 1.3%), type II (with one HSB, 0.07%), type IIIa (with two HSBs when THB is bigger, 28%), type IIIb (with two HSBs when FHB is bigger, 65.3%), and type IV (with three HSBs, 4.7%). There was no statistical difference between the left and right sides, except HVA, Meary, and pitch (*P* < 0.05); all a, b, c, d, and i have statistical difference between male and female (*P* < 0.05). Meanwhile, HVA and IMA and HVA and type group have a significant correlation. In summary, HVA and IMA and HVA and classification of HSBs have significant correlations. The classification and location of HSBs can be an important basis to choose operation methods and postoperation evaluation.

## 1. Introduction

Hallux valgus (HV) is a complex anatomical deformity that is challenging in treatment. Park et al. [1] pointed out that HV is a highly prevalent foot deformity that involved hallucal sesamoid bones (HSBs) and affected 23% of adults and 35.7% of elderly individuals. In the normal walking gait of a man, the hallucal sesamoid complex transmits 50% weight of the body and can transmit over 300% load during push-off, when the tibial side one undertakes the most part. That means HSBs play an important role in reducing load per unit area in the first metatarsal head, especially the tibial side one. Clinically, patients with sesamoid bones below the first metatarsal head were three to five times more likely to suffer HV than patients without sesamoid bone [2–5]. That means the classification of sesamoids may be a major factor affecting the occurrence and development of HV [6]. From this, it is necessary to learn about the relationship between classification of HSBs and HV severity which can be helpful to assist in clinical treatment and providing data indexes for postoperation evaluation.

In 1986, Mann and Coughlin [7] gave the first description of the severity of HV named Mann Type which included mild, moderate, and severe. The measurement data of HV are from weight-bearing X-rays. The imaging retrospective study of 500 cases of HV and 500 cases of non-HV foot in Theumann et al. [6] showed that the incidence of multiple seed bone in HV foot (32.3%) was significantly higher than that in non-HV foot (15.2%). Munuera et al. [8] found that there were significant differences between the HV angle of the multiseeded and nonfractionated feet by 474 foot imaging studies. Usually, there are two sesamoid below the head of the first metatarsal, the inner side one named tibial hallux sesamoid (THS) and the offside one named fibular hallux sesamoid (FHS); besides, THS is bigger than FHS [9–11]. However, all differences are only for the tibial bone, and the causal relationship between HSBs and HV is not clear now. Meanwhile, the “capstan mechanism” that exists in the first metatarsophalangeal joint will aggravate the first metatarsal varus and aggravate the relative subluxation of HSBs. So each classification of Mann Type touches upon HSBs subluxated, dislocation and a larger proximal articular set angle are obvious indicators for clinical operation [12–15]. To complete the data acquisition, a fitful method to measure the dislocation of seed bone is needed. By comparing, Hardy classification is the most incredible and rich method to tell the dislocation of seed bone [16]. However, Grade IV classification, which is based on Hardy classification, is more simple, practical, and fit for this research [17]. Clinically, the best choice for the treatment of HV is surgery. Different conditions always match with different solutions; surgical options for managing these problems include curettage, bone grafting, shaving, internal fixation, and partial or complete excision [18–21]. Thus, surgeons need to develop the best treatment plan through more data references. Anatomically, the sesamoid bone located at the muscle insertion between the tendon and the bone and is formed by tenostosis can change the angle of tendon insertion, enlarge the muscle power, and increase the muscular contractility. The stability of sesamoid is maintained by the ligament between them, the thickness of them, and the attachment of tendon. When the muscle group around them is stacked into power imbalance because of the surgery, worse pathology comes. Hence, it is necessary to learn the classification and location of HSBs, so that the relationship between the classification of HSBs and HV severity can be analyzed. The results can be an important basis to remind patients of prevention, help surgeons choose operation methods, and do the postoperation evaluation.

In general, the choice of HV surgery is the basic skill that the ankle surgeon needs to master. This study illustrated the relationship between the classification of HSB and HV severity to provide auxiliary parameters for ankle surgeons to select suitable surgical methods for patients. We measured 302 HSBs from 150 dry metatarsals from the Chinese to master the basic anatomical structure and morphology of the Chinese HSBs, which can improve the anatomical data of it and be helpful in clinical diagnosis and treatment.

## 2. Material and Methods

### 2.1. Materials

302 HSBs from 150 metatarsals which consist of 72 left and 78 right metatarsals were measured by X-ray and three-dimensional (3D) computed tomographic (CT) reconstruction techniques, which were weight-bearing, and recorded with an accuracy of up to 0.1 mm. All the 100 male and 50 female (mean 41 years, range 18–90 years) metatarsals belong to the Chinese Han nationality supposed by the Ethical Inspection Committee at Southwest Medical University (Luzhou, China).

### 2.2. Instruments

The X-ray and three-dimensional (3D) computed tomographic (CT) data of metatarsal were collected from digital radiography and a spiral CT scanner (Somatom Emotion; Siemens AG, Munich, Germany). Measurements were carried out by the software (UniReport version 2.0) of the PACS. Both the X-ray and CT scans were weight-bearing.

### 2.3. Method of Measure

**Figure 1:**
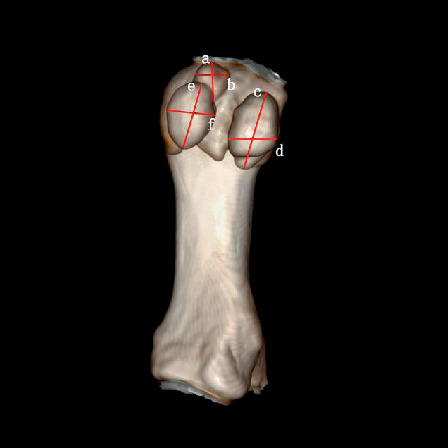
The long and short axis of the sesamoid bone. (a) The long axis of the medial distal sesamoid bone (the longest distance of the medial distal sesamoid bone). (b) The short axis of the medial distal sesamoid bone (the shortest distance of the medial distal sesamoid bone). (c) The long axis of the lateral distal sesamoid bone. (d) The short axis of the lateral distal sesamoid bone. (e) The long axis of the medial proximal sesamoid bone. (f) The short axis of the medial proximal sesamoid bone.

**Figure 2:**
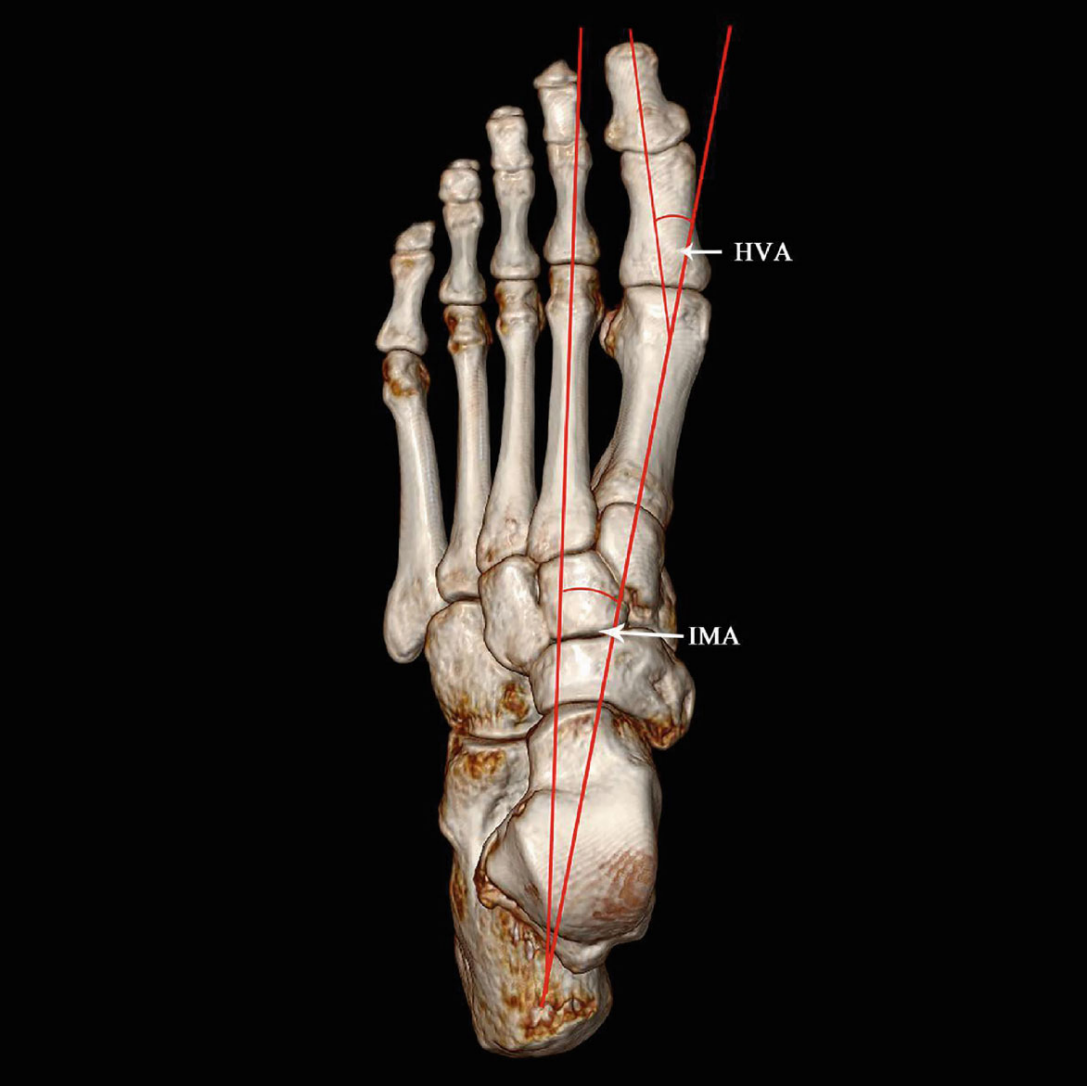
The angle to judge the severity of HV. On orthographic X-rays, the angle between the longitudinal axis of the first metatarsal bone and the longitudinal axis of the first phalangeal proximal phalangeal bone, normal < 16°. Intermetatarsal IMA: on the orthographic X-ray film, the angle between the longitudinal axis extension line of the first metatarsal bone and the second metatarsal bone, normal < 10°.

**Figure 3:**
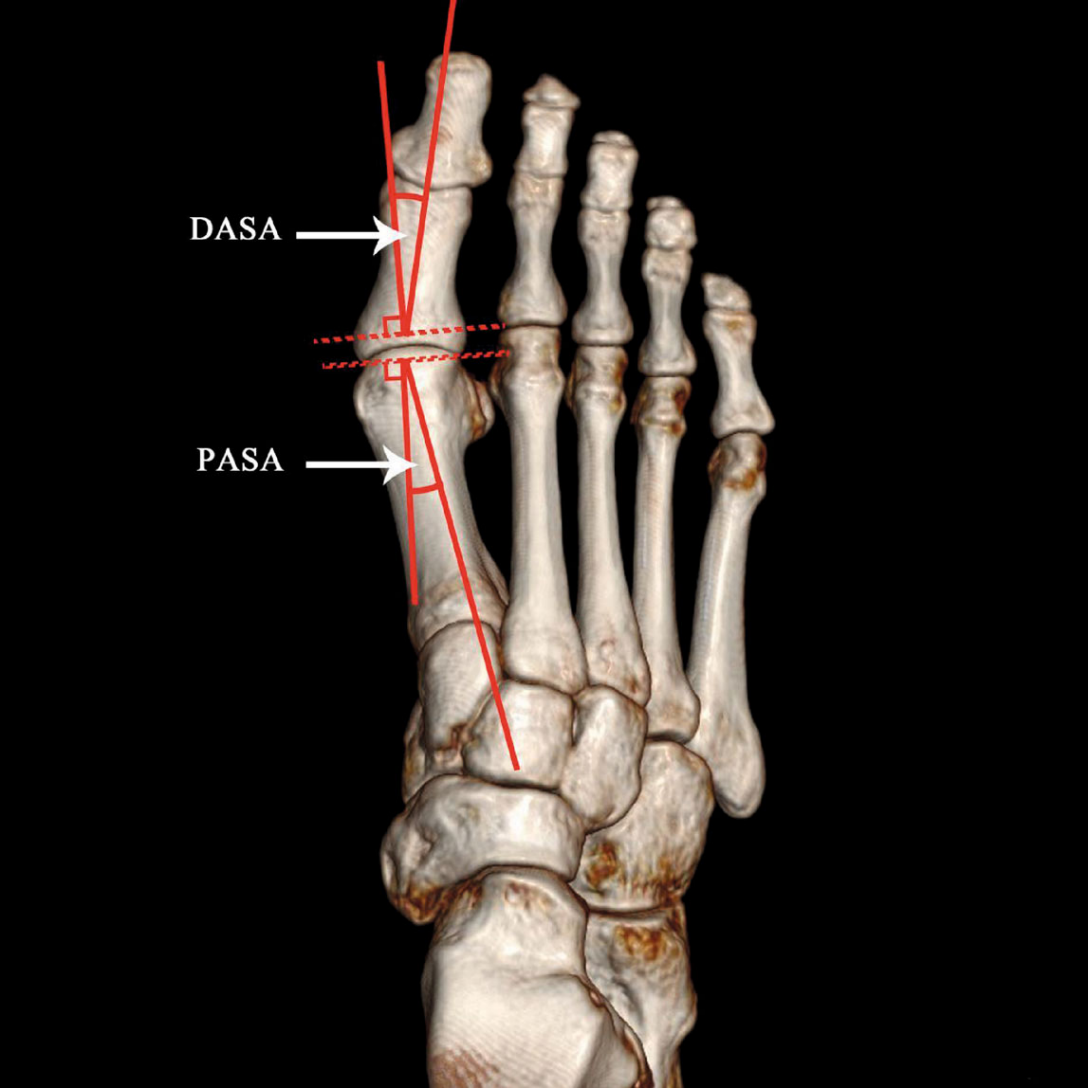
The angle to assist in judging the severity of HV. Distal articular set angle (DASA): on orthographic X-rays, the angle between the vertical line of the basal joint surface of the first phalangeal bone and the long axis of the first phalangeal bone, normal < 7°. Proximal articular set angle (PASA): on the orthographic X-ray film, the angle between the vertical line of the first metatarsal joint and the first metatarsal axis, normal < 10°.

1. a: the long axis of the medial distal sesamoid bone (the longest distance of the medial distal sesamoid bone)
2. b: the short axis of the medial distal sesamoid bone (the shortest distance of the medial distal sesamoid bone)
3. c: the long axis of the lateral distal sesamoid bone (the longest distance of the lateral distal sesamoid bone)
4. d: the short axis of the lateral distal sesamoid bone (the shortest distance of the lateral distal sesamoid bone)
5. e: the long axis of the medial proximal sesamoid bone (the longest distance of the medial proximal sesamoid bone)
6. f: the short axis of the medial proximal sesamoid bone (the shortest distance of the medial proximal sesamoid bone)
7. i: distance between medial and lateral sesamoid bones (Figure 1)
8. Degree of dislocation in sesamoid bone: taking the bisection line of the long axis of the first metatarsal axis as the reference line. Grade 0: no displacement of medial sesamoid relative to reference line; Grade 1: overlap of less than 50% of medial sesamoid to reference line; Grade 2: overlap of greater than 50% of medial sesamoid to reference line; Grade 3: complete displacement of medial sesamoid beyond reference line laterally
9. Hallux valgus angle (HVA): on orthographic X-rays, the angle between the longitudinal axis of the first metatarsal bone and the longitudinal axis of the first phalangeal proximal phalangeal bone, normal < 16°
10. Intermetatarsal angle (IMA): on the orthographic X-ray film, the angle between the longitudinal axis extension line of the first metatarsal bone and the second metatarsal bone, normal < 10° (Figure 2)
11. Proximal articular set angle (PASA): the angle between the vertical line of the first metatarsal joint and the first metatarsal axis, normal < 10°
12. Distal articular set angle (DASA): on orthographic X-rays, the angle between the vertical line of the basal joint surface of the first phalangeal bone and the long axis of the first phalangeal bone, normal < 7° (Figure 3)
13. Angle Meary: the first metatarsal angle of the talus
14. Angle pitch: calcaneal inclination angle

Inclusion criteria are as follows: (1) the patients older than 18 years who had weight-bearing X-rays (AP and lateral) and CT of the same foot with both studies obtained of each other and (2) no history of the first metatarsophalangeal joint operation.

At the same time, the metatarsus had to be ruled out because of these situations: (1) trauma history of injury to the first metatarsophalangeal joint and (2) with osteoarthropathy of the first metatarsophalangeal joint, including osteoarthritis, tumor, and infection.

To ensure the evaluation of interobserver reliability, all subjects were carefully observed by the same investigator twice, who is a general orthopedic surgeon engaged in anatomical work for more than 5 years. Then, take the mean (accurate to 0.1 mm).

### 2.4. Statistical Analysis

The parameters were expressed as the mean ± standard deviation (SD). A statistical analysis was performed using SPSS, version 20.0 (IBM Corp., Armonk, NY, USA). The normality tests were used to check the variables studied, and the variables conformed to the normal distribution. Independent samples *t*-test was applied to identify differences between measurements in male patients and female patients. All hypothesis tests were implemented by adopting a 5% significance level, and *P* values equal to or smaller than 0.05 were considered statistically significant. Bivariate correlation tests the relationship between two variables shown by a simple scatter plot. *α* ≤ 0.05 is taken as the test level to tell if there is a significant correlation.

## 3. Results

**Figure 4:**
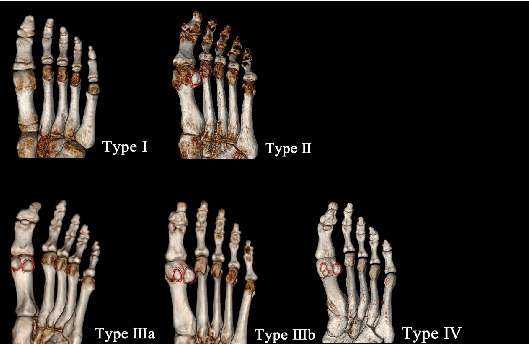
The types of HSBs on inferior by 3D CT image. Type I: no HSB; type II: with one HSB; type IIIa: with two HSBs when THB is bigger; type IIIb: with two HSBs when FHB is bigger; and type IV: with three HSBs. All HSBs are circled in red.

In this study, a total of 14 qualitative features were evaluated on X-ray and 3D CT (by 3 trainees, respectively) among patients. All data were normally distributed. Meanwhile, the first metatarsophalangeal joint is divided into four different types: type I (no HSB, 1.3%), type II (with one HSB, 0.07%), type IIIa (with two HSBs when THB is bigger, 28%), type IIIb (with two HSBs when FHB is bigger, 65.3%), and type IV (with three HSBs, 4.7%) (Figure 4). Regarding HVA in these types, there are 141 cases (94%) with mild HV, 9 cases (6%) with moderate HV, and no case (0%) with severe HV. Regarding IMA in these types, there are 146 cases (97.3%) with mild HV, 4 cases (2.7%) with moderate HV, and no case (0%) with severe HV (Table 1). Moreover, Table 2 lists the frequency of medial HSB displace grade found in 3D CT images. There are 46 (30.7%) cases of Grade 0 dislocation, 57 (38%) cases of Grade 1 dislocation, 43 (28.7%) cases of Grade 2 dislocation, 43 (28.7%) cases of Grade 3 dislocation, and 4 (2.7%) cases of Grade 4 dislocation (Table 1). In Table 2, there are statistical differences between the dislocation and classification of the sesamoids (*P* < 0.05), which means type IIIa and type IIIb caught worse dislocation than other types, especially type IIIb.

The measurements of the length and angle on sesamoid bones and the first metatarsophalangeal joint are listed in Table 3 and Table 4. Table 3 illustrates whether there are statistical differences between the left and right sides of all data, when Table 4 illustrates whether there are statistical differences between the male and female of all data. Among those, HVA, angle Meary, and angle pitch have statistical differences between left and right which are listed in Table 3 (*P* < 0.05). Also, all a, b, c, d, and i have statistical differences between male and female which are listed in Table 4 (*P* < 0.05). There is no statistical difference at angle between male and female. For qualitative assessments on X-ray and CT, interreader reliability was good to excellent for all features (Table 5 and Table 6).

**Figure 5:**
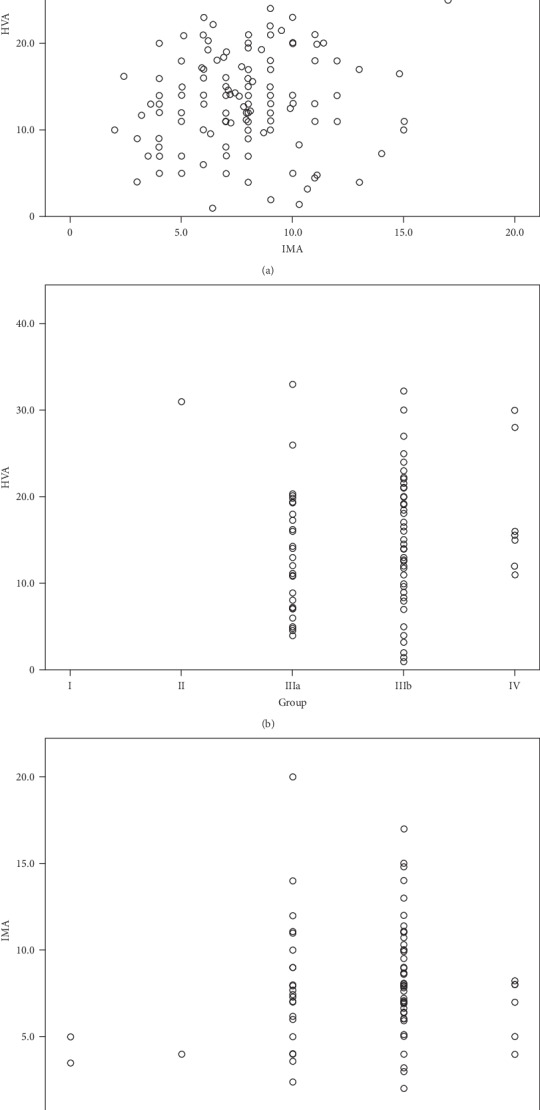
(a) Relationship between HVA and IMA. ∗*P* < 0.05, *R* = 0.422. Bigger HVA is accompanied by bigger IMA most of the time. (b) Relationship between HVA and the classification of HSBs. ∗*P* < 0.05, *R* = 0.208. Patients with type IIIa and type IIIb sesamoid bones have a higher probability of HV. (c) Relationship between IMA and the classification of HSBs. ∗*P* > 0.05, *R* = 0.107. There is no significant correlation between IMA and type group.

Comparing the relationship among HVA, IMA, and the classification of HSBs, HVA and IMA and HVA and the classification of HSBs have significant correlation. Type IIIb has a greater chance of catching HV than type I, type II, type IIIa, and type IV. These relationships are showed by a simple scatter plot and presented in Figures 5(a)–5(c).

## 4. Discussion

Thus far, physical examination, radiographs, and other specialized studies assist with the classification of sesamoid pathology [7, 8]. And the main treatment about HV is surgery which includes tissue surgery and osseous surgery. At the beginning, most of the initial treatment involves an expectant treatment named accommodative orthosis, but surgical intervention has to be chosen in recalcitrant cases.

In this research, 150 metatarsals were measured by X-ray and three-dimensional (3D) computed tomographic (CT) reconstruction technique. The result can be seen that bipartite sesamoid bones are the largest number of types which covered 93.3%. Among these, type IIIb (with two HSBs when FHB is bigger, 65.3%) covered more than type IIIa (with two HSBs when THB is bigger, 28%). Meanwhile, absence and three-seed bone also exist. Except that, the statistical analysis demonstrated that there is no statistical difference between left and right sides, except that HVA was 15.989 ± 6.244 mm on the left and 12.179 ± 5.844 mm on the right (*P* < 0.001), Meary was 15.256 ± 11.384 mm on the left and 24.545 ± 10.303 mm on the right (*P* < 0.001), and pitch was 30.644 ± 6.194 mm on the left and 27.705 ± 6.275 mm on the right (*P* < 0.001). When the HVA on the left side is bigger than that on the right, bivariate correlation tests the relationship among HVA, IMA, and the classification of HSBs; the result demonstrated that HVA and IMA and HVA and the classification of HSBs have significant correlation, which means patients with bipartite sesamoid bones have a higher probability of HV and bigger HVA is accompanied by bigger IMA most of the time. Moreover, there are statistical differences that a was 12.077 ± 1.824 mm on male and 11.161 ± 1.932 mm on female (*P* < 0.05), b was 8.344 ± 1.389 mm on male and 7.888 ± 1.116 mm on female (*P* < 0.05), c was 12.227 ± 1.161 mm on male and 11.276 ± 1.508 mm on female (*P* < 0.05), d was 9.116 ± 1.169 mm on male and 8.561 ± 0.971 mm on female (*P* < 0.05), and i was 2.421 ± 1.071 mm on male and 1.756 ± 0.992 mm on female (*P* < 0.05). Some study pointed that the shape of the metatarsal head may be a cause of HV which means there are some differences about anatomic characteristics like those [19–21] such as the anatomic differences between type IIIa and type IIIb.

Moreover, when it comes to type, IIIa and IIIb caught worse dislocation than other types, especially type IIIb. The number of type may be a factor, the relationship between HVA and the classification of HSBs may be another. The latter can also be related to the anatomic structure. HV deformity consists of lateral deviation of the proximal phalanx on the metatarsal head with accompanying medial deviation of the first metatarsal which leaded to an enlargement of the medial bony eminence of the first metatarsal head [22–24]. As the deformity progresses, the medial bony eminence of the first metatarsal head can be accentuated with bursal thickening or inflammatory bursitis. Although pain will not occur to every patient and is not proportional to deformity, some soft tissues in osseous are prone to acute pollicis bursal inflammation caused by long-term compression and friction. Long-term anomalies can lead to bias, oblique span, and even osteoarthritis which seriously affect the quality of life of patients which choose to seek medical treatment. Moreover, the injury of sesamoid includes sesamoid fracture (SF), sesamoid dislocation (SD), sesamoiditis, sesamoid osteomyelitis, and sesamoid tuberculosis. In summary, it is typically caused by a repetitive stress injury [2, 23–25].

In clinic, patients with sesamoid bone below the first metatarsal head were three to five times more likely to suffer HV, and Chevron Akin Osteotomy is one of the most widely studied and widely used kinds of research [2–5, 26–31]. The measurement about 302 HSBs on 150 dry metatarsals from Chinese can help the clinician to master the basic anatomical structure and morphology of the Chinese HSBs, which can improve the anatomical data of it and be helpful in clinical diagnosis and treatment. Though there are many researches about HV or anatomic characteristic, hardly any research studied the relationship between them, including the classification.

Nevertheless, there are still some shortages. Firstly, though some classifications were listed here to help us do better in clinic, the sesamoid bones have a complex structure and the appearance of new type cannot be assured. Secondly, the samples in this research are limited to the southwest and the sample size is not so adequate now. Thirdly, the HSBs of some minors are not completely ossified and are not taken into account.

## 5. Conclusion

In summary, HVA and IMA and HVA and the classification of HSBs have a significant correlation. Type IIIb has a greater chance of catching HV than type I, type II, type IIIa, and type IV. Hence, the classification and location of HSBs can be an important basis to choose operation methods and provide data index for postoperation evaluation.

## Figures and Tables

**Table 1:** Frequency of HVA and IMA severity in each classification found in 3D CT images.

	HVA (°)			IMA (°)		
	Mild	Moderate	Severe	Mild	Moderate	Severe
I	0	0	0	0	0	0
II	0	1	0	1	0	0
IIIa	40	2	0	41	1	0
IIIb	94	4	0	95	3	0
IV	5	2	0	7	0	0

Note: mild (grade) in HVA is less than 25 degrees, moderate (grade) in HVA is between 25 and 35 degrees, and severe (grade) in HVA is more than 35 degrees; mild (grade) in IMA is less than 15 degrees, moderate (grade) in IMA is between 15 and 20 degrees, and severe (grade) in IMA is more than 20 degrees.

**Table 2:** Frequency of medial HSB displace grade found in 3D CT images.

		Dislocation∗			
		Grade 0	Grade 1	Grade 2	Grade 3
Classification	I	—	—	—	—
	II	—	—	—	1
	IIIa	10	19	12	1
	IIIb	31	36	29	2
	IV	3	2	2	—

∗*P* < 0.05 vs. classification. Note: taking the bisection line of the long axis of the first metatarsal axis as the reference line: Grade 0: no displacement of sesamoid relative to the reference line; Grade 1: overlap of less than 50% of sesamoid to the reference line; Grade 2: overlap of greater than 50% of sesamoid to the reference line; and Grade 3: complete displacement of medial sesamoid beyond the reference line laterally.

**Table 3:** Mean values of measurements of sesamoid bones and the first metatarsophalangeal joint (mean ± SD).

	Term	Total	Left	Right	*P* value
Distance (mm)	a	11.765 ± 1.905	12.083 ± 1.854	11.465 ± 1.916	0.324
	b	8.198 ± 1.354	8.117 ± 1.311	8.269 ± 1.395	0.391
	c	11.882 ± 1.361	12.106 ± 1.145	11.692 ± 1.501	0.205
	d	8.948 ± 1.132	9.059 ± 1.198	8.851 ± 1.070	0.379
	e	8.478 ± 2.646	9.350 ± 2.224	7.925 ± 3.352	0.590
	f	7.400 ± 2.196	8.425 ± 1.931	5.433 ± 1.328	0.454
	i	2.161 ± 1.066	2.339 ± 1.130	2.199 ± 0.994	0.222
Angle (°)	HVA	14.008 ± 6.314	15.989 ± 6.244	12.179 ± 5.844	0.001∗
	IMA	7.911 ± 2.924	8.224 ± 3.002	7.623 ± 2.839	0.211
	PASA	5.244 ± 3.472	5.452 ± 3.394	5.812 ± 3.275	0.566
	DASA	5.011 ± 4.117	5.554 ± 4.466	6.494 ± 4.959	0.17
	Meary	18.779 ± 11.841	15.256 ± 11.384	24.545 ± 10.303	0.001∗
	Pitch	29.529 ± 6.361	30.644 ± 6.194	27.705 ± 6.275	0.001∗

∗*P* < 0.001 vs. left. Note: a total of 150 images (72 left, 78 right) have been used for this table. The mean long axis and short axis of each HB: a, b, c, d, e, and f; the mean distance between medial and lateral sesamoid bones: i; some mean angles on the first metatarsophalangeal joint: HVA, IMA, PASA, and DASA; the mean angel of instep: Meary and pitch.

**Table 4:** Mean values of measurements of sesamoid bones and the first metatarsophalangeal joint (mean ± SD).

	Term	Total	Male	Female	*P* value
Distance (mm)	a	11.765 ± 1.905	12.077 ± 1.824	11.161 ± 1.932	0.010∗
	b	8.198 ± 1.354	8.344 ± 1.389	7.888 ± 1.116	0.019∗
	c	11.882 ± 1.361	12.227 ± 1.161	11.276 ± 1.508	0.001∗
	d	8.948 ± 1.132	9.116 ± 1.169	8.561 ± 0.971	0.002∗
	i	2.161 ± 1.066	2.421 ± 1.071	1.756 ± 0.992	0.001∗
Angle (°)	HVA	14.008 ± 6.314	13.307 ± 6087	15.410 ± 6.602	0.062
	IMA	7.911 ± 2.924	7.783 ± 2.872	8.168 ± 3.038	0.458
	PASA	5.244 ± 3.472	6.053 ± 3.297	4.972 ± 3.426	0.217
	DASA	5.011 ± 4.117	6.128 ± 4.814	5.096 ± 4.177	0.296
	Meary	18.779 ± 11.841	18.274 ± 12.348	19.816 ± 10.808	0.375
	Pitch	29.529 ± 6.361	29.864 ± 6.183	28.842 ± 6.744	0.621

∗*P* < 0.05 vs. male. Note: a total of 150 images (100 male, 50 female) have been used for this table. The mean long axis and short axis of each HB: a, b, c, d, e, and f; the mean distance between medial and lateral sesamoid bones: i; some mean angles on the first metatarsophalangeal joint: HVA, IMA, PASA, and DASA; the mean angel of instep: Meary and pitch.

**Table 5:** Interreader reliability on X-ray.

Measurements	Kappa (95%) ∗weighted kappa
Hallux valgus angle	0.92 (0.88, 0.96)
Intermetatarsal angle	0.86 (0.8, 0.94)
Proximal articular set angle	0.71 (0.53, 0.87)
Distal articular set angle	0.67 (0.58, 0.86)
Angle Meary	0.62 (0.46, 0.87)
Angle pitch	0.67 (0.46, 0.82)

**Table 6:** Interreader reliability on CT.

Measurements	Kappa (95%) ∗weighted kappa
The long axis of the medial distal sesamoid bone	0.75 (0.62, 0.9)
The short axis of the medial distal sesamoid bone	0.71 (0.65, 0.82)
The long axis of the lateral distal sesamoid bone	0.81 (0.75, 0.96)
The short axis of the lateral distal sesamoid bone	0.62 (0.47, 0.98)
The long axis of the medial proximal sesamoid bone	0.77 (0.62, 0.96)
The short axis of the medial proximal sesamoid bone	0.74 (0.61, 0.89)
Distance between medial and lateral sesamoid bones	0.8 (0.76, 0.99)
Degree of dislocation in sesamoid bone	1 (1, 1)

## Data Availability

The related data used to support the findings of this study are restricted by the medical ethics committee of School of Basic Medical Sciences, Southwest Medical University. Data are available from Lei Zhang (email: zhanglei870722@126.com) for researchers who meet the criteria for access to confidential data.
